# Comparison of Deep Learning Architectures for Cardiac Contour Segmentation in Catheterization Radiographs

**DOI:** 10.7759/cureus.103341

**Published:** 2026-02-10

**Authors:** Kian A Huang, Bharath Subramanian, Haris K Choudhary, Aziz U Rehman, Neelesh S Prakash

**Affiliations:** 1 Radiology, University of South Florida Morsani College of Medicine, Tampa, USA

**Keywords:** ai, computer vision, deep learning, image segmentation, unet

## Abstract

Introduction

Accurate cardiac image segmentation is essential for quantitative assessment of cardiac anatomy and function. Manual segmentation, while considered the reference standard, is time-consuming and subject to inter- and intra-observer variability. Deep learning (DL) models, particularly convolutional neural networks (CNNs), have shown promise for automating this process. Among these, U-Net and DeepLabV3 are widely used architectures; however, direct comparisons between them for cardiac silhouette segmentation on cardiac catheterization radiographs are limited.

Methods

A supervised DL study was conducted using 1717 anonymized chest radiographs during cardiac catheterization with corresponding binary segmentation masks of the cardiac silhouette. Images were resized to 256 × 256 pixels, normalized, and divided into training (70%), validation (20%), and test (10%) sets. Two segmentation models were implemented: (1) a modified U-Net with batch normalization and dropout regularization, and (2) a DeepLabV3 network with a MobileNetV2 backbone pretrained on ImageNet. Both models were trained using the Adam optimizer and binary cross-entropy loss with early stopping based on validation performance. Segmentation performance was evaluated on the test set using the Dice similarity coefficient, Intersection over Union (IoU), and pixel accuracy. Statistical comparisons were performed using the Wilcoxon signed-rank test, and effect sizes were calculated using the rank-biserial correlation (RBC).

Results

U-Net consistently outperformed DeepLabV3 across all evaluation metrics. U-Net achieved a mean Dice of 0.9454, IoU of 0.8980, and pixel accuracy of 0.9844, compared to DeepLabV3 (Dice = 0.9321, IoU = 0.8742, pixel accuracy = 0.9806). These differences were statistically significant (Dice: W = 2849.0, p < 1 × 10⁻¹², RBC = 0.617; IoU: W = 2810.0, p < 1 × 10⁻¹², RBC = 0.622; pixel accuracy: W = 2768.5, p < 1 × 10⁻¹², RBC = 0.628), indicating large effect sizes favoring U-Net.

Conclusions

U-Net achieved significantly higher segmentation accuracy than DeepLabV3 for cardiac silhouette segmentation on catheterization radiographs. Its encoder-decoder architecture with skip connections enabled superior boundary delineation, reaffirming U-Net as a strong baseline for medical image segmentation. Automated cardiac silhouette segmentation may enhance the efficiency and reproducibility of cardiothoracic ratio estimation, cardiomegaly detection, and longitudinal cardiac monitoring. Future studies should focus on multi-institutional validation, chamber-level segmentation, and benchmarking against transformer-based architectures to advance clinical integration.

## Introduction

Cardiovascular diseases (CVDs) are currently the leading cause of death, morbidity, and mortality worldwide, according to the World Health Organization. An estimated 19.8 million people died from CVDs in 2022, with 85% of these deaths being attributed to stroke and heart attacks [1]. This clearly underscores the need for accurate diagnostic and prognostic tools. Among the various imaging techniques available for cardiovascular assessment, chest radiography remains one of the most widely used modalities due to its accessibility, low cost, and rapid acquisition, making it particularly valuable in both routine screening and acute care settings. Chest radiographs allow for non-invasive visualization of the cardiac silhouette and surrounding thoracic structures, enabling clinicians to assess overall heart size, detect cardiomegaly, evaluate pulmonary vasculature, and identify signs of heart failure or pericardial effusion. A core component of many clinical assessments is the segmentation of the cardiac silhouette from these radiographic images. Cardiac silhouette segmentation involves delineating the heart border from the surrounding lung fields and mediastinal structures, from which clinically relevant measurements such as the cardiothoracic ratio (CTR) - a key indicator of cardiac enlargement - can be obtained. Unlike advanced cross-sectional imaging modalities that enable chamber-specific analysis, chest radiographs primarily provide an assessment of the overall cardiac silhouette rather than individual cardiac chambers or vessels, yet this information remains clinically valuable for initial evaluation and monitoring of cardiac disease [2].

Traditionally, cardiac image segmentation has a manual delineation performed by cardiologists and radiologists. Even though this may be the reference standard, the manual approaches are time-intensive and prone to significant inter- and intra-observer variability, especially in cases of complex anatomy or poor image quality [3]. As a result, the variability limits reproducibility and scalability, which has created a pressing demand for automated and clinically usable segmentation methods. Across the past decade, automated segmentation techniques have been explored utilizing conventional techniques such as active contours, atlas-based registration, and statistical shape models, which have all demonstrated some utility in cardiac segmentation [4]. However, these techniques have often struggled when tasked with handling heterogeneous datasets, diverse imaging protocols, and the wide variability of cardiac pathologies. For these techniques to achieve acceptable accuracy, they often require feature engineering or prior knowledge [2]. In contrast, the advance of deep learning (DL) has marked a shift in medical image analysis. DL-based models are inherently good at automatically identifying complex features from data for object detection and segmentation. These characteristics are acquired directly from data through a general learning method and in an end-to-end manner. This allows DL-based algorithms to be easily utilized in other image analysis tasks, such as cardiac segmentation.

Convolutional neural networks (CNNs) are a specialized type of deep learning model that are designed to process visual information by emulating how the human visual system interprets images. This is achieved through the employment of networks that are made up of several layers that can gradually extract increasingly more complex features, starting with basic edges and textures in the initial layers, which are subsequently merged into more advanced patterns such as shapes and anatomical structures in the deeper layers [5]. The ability of CNNs to automatically learn hierarchical image features directly from data has eliminated the need for any descriptors and allows for the robust generalization across various patient populations. Within CNN-based architectures, encoder-decoder models have demonstrated significant potential since they can grasp both overarching contextual information and intricate spatial details necessary for accurate boundary delineation.

The most well-known and commonly used encoder-decoder network for biomedical image segmentation is U-Net. This network's symmetric encoder-decoder architecture with skip connections allows for the preservation of high‑resolution spatial features from the encoder so it can be integrated into the decoder, improving boundary delineation and segmentation accuracy [6]. Specifically, U‑Net employs these skip connections between the encoder and decoder to recover spatial context lost in the down-sampling path, allowing for more precise results. U-Net's simplicity, efficiency, and adaptability have made it the baseline architecture for medical image segmentation challenges, which include the Automated Cardiac Diagnosis Challenge dataset, where U‑Net and its derivatives consistently achieved top performance. Specifically, U‑Net‑based methods achieved Dice scores in the ~0.90‑0.95 range for segmenting left and right ventricles and myocardium, and about 0.97 correlation between automatic vs expert clinical indices [7].

DeepLabV3 is another DL-based semantic segmentation model that is useful for tasks requiring multiscale contextual reasoning. It is built upon an encoder-decoder structure where DeepLabV3 leverages atrous convolution to expand the effective receptive field without increasing the number of parameters or reducing spatial resolution, which enables dense predictions with enhanced contextual awareness [8]. A key component is the Atrous Spatial Pyramid Pooling module, which applies multiple parallel atrous convolutions at differing dilation rates in order to capture image context at multiple scales efficiently. DeepLabV3 can be further enhanced when coupled with a lightweight backbone network such as MobileNetV2, which introduces inverted residual blocks and linear bottlenecks to improve feature representation while minimizing model size [9]. Ultimately, the combination of atrous convolutions and MobileNetV2's efficient architecture allows DeepLabV3 to maintain competitive segmentation accuracy with significantly fewer parameters and lower latency, which is particularly suitable for deployment in resource-constrained or real-time clinical imaging environments.

Despite their popularity, direct head-to-head comparisons of U-Net and DeepLabV3 on cardiac imaging datasets remain limited. Evaluating these models on the same dataset and under comparable conditions can help clarify their respective strengths, limitations, and suitability for clinical deployment. The present study aims to compare the performance of U-Net and DeepLabV3 on a curated cardiac image segmentation dataset. By examining standard metrics such as Dice similarity coefficient, Intersection over Union (IoU), and pixel accuracy, we provide insight into how these architectures perform in segmenting cardiac structures.

## Materials and methods

Dataset and preprocessing 

We conducted a supervised deep learning project using 1717 anonymized cardiac catheterization radiographs with corresponding binary segmentation masks of the cardiac silhouette. Images were retrieved from a publicly available dataset on Kaggle ("Cardiac Semantic Segmentation Dataset") available under CC BY-SA 4.0 license, which did not include any patient demographic or institutional information. All images and masks were stored in matched directories and preprocessed prior to model training. Each radiograph was resized to 256 × 256 pixels with cropping to preserve the aspect ratio and normalized to values between 0 and 1. The corresponding masks were resized using nearest-neighbor interpolation to preserve boundary sharpness, and binarized at a threshold of 127 to ensure that all pixel values were encoded as either foreground (cardiac contour) or background.

The dataset was divided into training, validation, and test sets in a 70:20:10 ratio using a randomized split with a fixed seed for reproducibility. Images and masks were linked in paired fashion to maintain alignment, and batches of 16 images were created for training. Data loading and preprocessing were handled using the TensorFlow data pipeline to allow efficient shuffling, batching, and prefetching.

Model architectures and training

**Figure 1:**
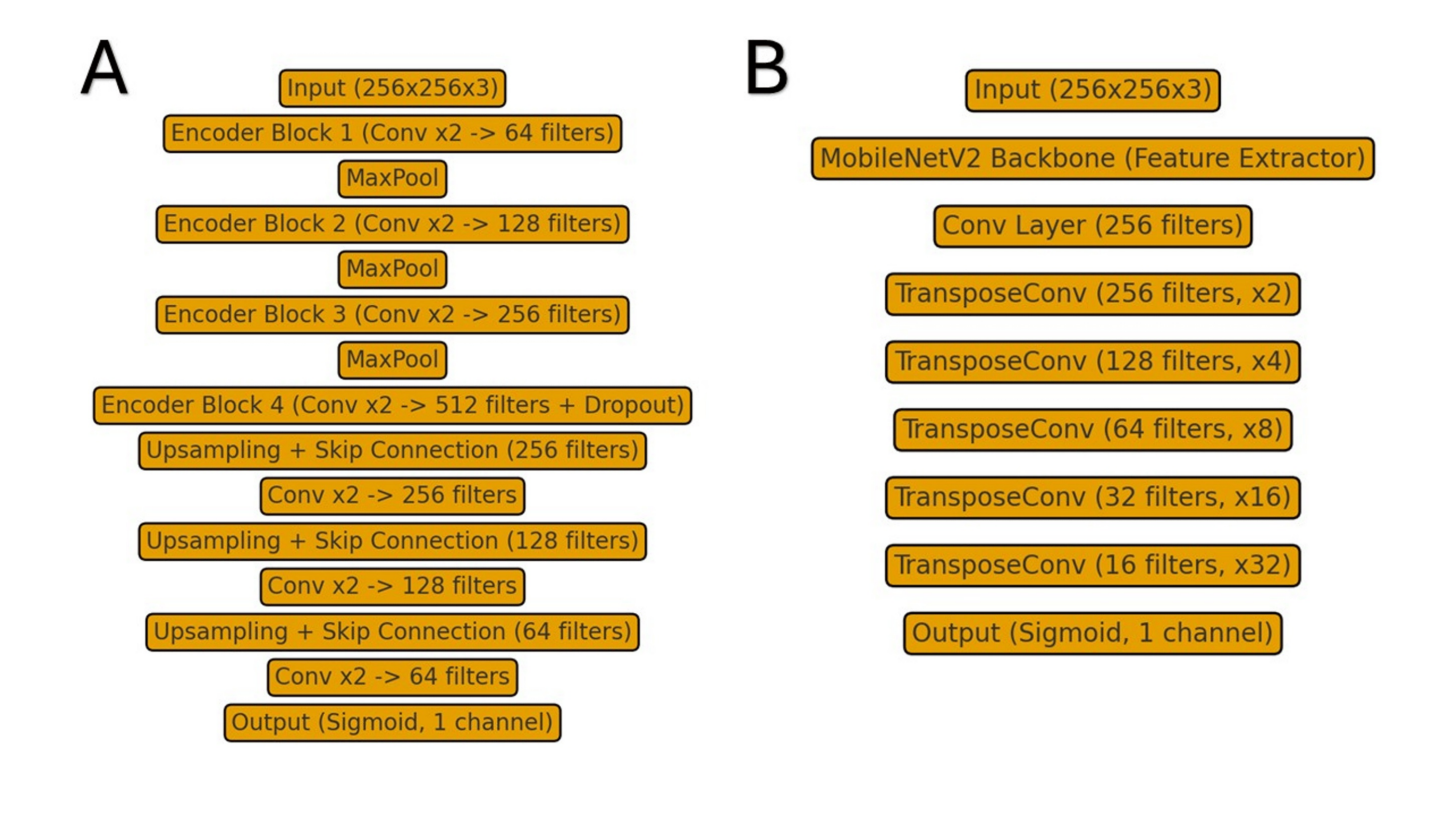
Comparison of segmentation architectures A) U-Net architecture showing encoder-decoder structure with skip connections; B) DeepLabV3-based model using a MobileNetV2 backbone with progressive upsampling. Both output a single-channel sigmoid mask for binary segmentation.

Two segmentation architectures were implemented and compared. The first was a standard U-Net with an encoder-decoder structure and skip connections, which was modified to include batch normalization at each convolutional block and dropout in the bottleneck layer to reduce overfitting. The second was an original DeepLabV3 model with a MobileNetV2 backbone pretrained on ImageNet, where the first 100 layers were frozen, and the remaining layers were fine-tuned. This configuration was determined empirically through preliminary experiments, where freezing these early layers resulted in improved validation performance compared to fine-tuning the entire network. In both models, the output layer consisted of a single channel with a sigmoid activation function to generate pixel-level probability maps. Architectures of each model can be seen in Figure 1.

Models were compiled using the Adam optimizer with a learning rate of 0.001 and binary cross-entropy loss. The batch size was set to 16 for both models. Training was performed for up to 100 epochs with early stopping applied if the validation loss failed to improve for seven consecutive epochs. A learning rate reduction on a plateau was implemented, reducing the learning rate by a factor of 0.5 after three epochs without improvement. Model checkpoints were saved at the epoch of the best validation performance.

Statistical methods

Evaluation was performed on the held-out test set. Model performance was quantified using three complementary metrics: the Dice similarity coefficient, Intersection over Union (IoU), and pixel accuracy. The Dice coefficient measures the degree of overlap between the predicted segmentation and the ground truth mask, with values ranging from 0 (no overlap) to 1 (perfect overlap); it is especially sensitive to how well the model captures the boundaries of the cardiac contour. IoU, also called the Jaccard index, similarly measures overlap but penalizes both false positives and false negatives more strongly, providing a stricter assessment of segmentation quality. Pixel accuracy reflects the proportion of correctly classified pixels across the entire image, including both cardiac and non-cardiac regions, and therefore captures overall correctness but can be influenced by the large background area in catheterization radiographs. All three metrics were computed for each image and then averaged across the test set to provide summary performance values.

To compare segmentation performance between models, we performed Wilcoxon signed-rank tests on the paired per-image Dice, IoU, and pixel accuracy values. This nonparametric test was chosen because it does not assume normally distributed differences and is well-suited for paired data. In addition, we computed effect sizes using the rank-biserial correlation (RBC), which quantifies the magnitude of differences between models beyond statistical significance.

To complement hypothesis testing, effect sizes were computed using the RBC, which quantifies the magnitude and direction of model differences. Statistical significance was set at p < 0.05. All analyses were performed using Python (version 3.13).

## Results

Both segmentation models were trained with early stopping protocols for 23 epochs on the training dataset and subsequently evaluated on the independent held-out test set. U-Net consistently outperformed DeepLabV3 across all three primary evaluation metrics: Dice similarity coefficient, IoU, and pixel-level accuracy. On average, U-Net achieved a Dice of 0.9454, IoU of 0.8980, and pixel accuracy of 0.9844, whereas DeepLabV3 achieved a Dice of 0.9321, IoU of 0.8742, and pixel accuracy of 0.9806.

As seen in Table 1, statistical testing using the Wilcoxon signed-rank test confirmed that these differences were significant for all three metrics. Dice (W = 2849.0, p < 0.001, RBC = 0.617), IoU (W = 2810.0, p < 0.001, RBC = 0.622), and pixel accuracy (W = 2768.5, p < 0.001, RBC = 0.628) all favored Model A, with large effect sizes. Positive RBC values indicate that U-Net outperformed DeepLabV3 across nearly all paired samples. These findings highlight a robust and consistent improvement in segmentation performance with U-Net relative to DeepLabV3.

It should be noted that pixel accuracy values are inherently high in cardiac silhouette segmentation due to the large proportion of background pixels correctly classified as background; therefore, Dice and IoU provide more meaningful assessments of segmentation quality by focusing specifically on boundary delineation performance.

**Table 1:** Segmentation test metrics Segmentation performance metrics derived from the hold-out test set for U-Net and DeepLabV3

Metric	U-Net (mean)	DeepLabV3 (mean)	Wilcoxon statistic	p-value	Effect sSize
Dice	0.9454	0.9321	2849.0	< 0.001	0.617
Intersection over Union	0.8980	0.8742	2810.0	< 0.001	0.622
Pixel accuracy	0.9844	0.9806	2768.5	< 0.001	0.628

**Figure 2:**
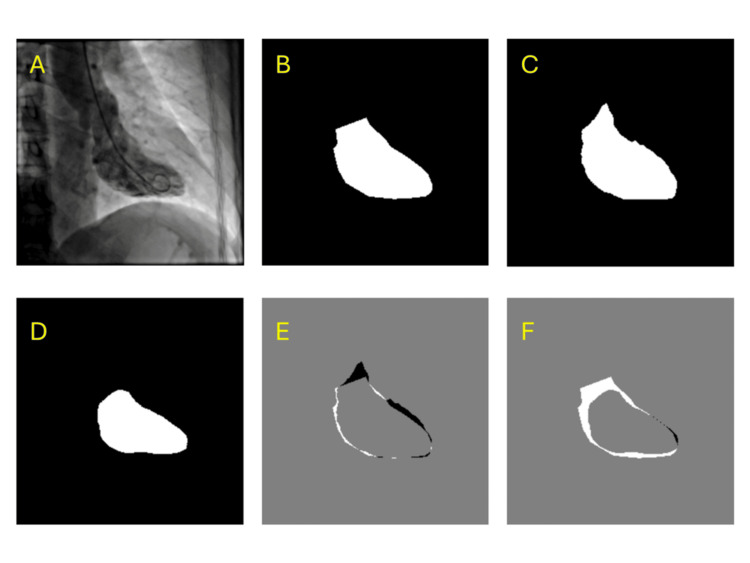
Example segmentation results Panels show (A) source image, (B) ground truth mask, (C) U-Net prediction, (D) DeepLabV3 prediction, (E) difference map of U-Net vs ground truth, and (F) difference map of DeepLabV3 vs ground truth.

Qualitative comparisons and sample test images are shown in Figure 2. Each figure panel displays the original source image, the ground truth segmentation mask, the U-Net prediction, the DeepLabV3 prediction, the difference map between U-Net and ground truth, and the difference map between DeepLabV3 and ground truth. Visual inspection further illustrates that U-Net achieved closer alignment with the ground truth masks, with smaller and more localized error regions compared to DeepLabV3.

## Discussion

In this study, U-Net consistently outperformed DeepLabV3 in the segmentation of the cardiac silhouette on catheterization radiographs, achieving higher Dice, IoU, and pixel accuracy scores with statistically significant and large effect sizes. These findings reaffirm U-Net's suitability not only as a benchmark architecture but also as a practical candidate for potential future integration. In particular, automated cardiac silhouette segmentation could streamline cardiothoracic ratio assessment in high-volume chest radiograph settings, reducing inter-reader variability and enabling faster triage. This technical demonstration underscores that even widely studied models such as U-Net can have direct utility when applied to task-specific clinical contexts. By contrast, DeepLabV3, while advantageous for tasks requiring multiscale contextual reasoning, may be less well suited for the relatively localized and anatomically constrained region of the cardiac contour. Recent systematic reviews have highlighted U-Net's prominence in medical image segmentation, with U-Net variants consistently ranking among the top-performing architectures in cardiac segmentation tasks across multiple modalities [8]. U-Net-based models have achieved mean Dice scores exceeding 0.85 for atrial and ventricular segmentation in cardiac MRI and CT datasets, while also performing competitively across echocardiography segmentation benchmarks [2]. In contrast, studies implementing DeepLabV3 in medical imaging have demonstrated its success in natural image segmentation tasks, though its specific application in cardiac catheterization radiograph segmentation remains limited. DeepLabV3+ has achieved Dice scores of 0.88 in modified architectures for COVID-19 lung segmentation in CT images, suggesting that the architecture's potential varies significantly with the specific anatomical target [10].

The performance gap was most pronounced for Dice and IoU, whereas pixel accuracy showed only modest differences between the models. This reflects the limitation of accuracy-based metrics in highly imbalanced images such as chest radiographs, where large background regions dominate. Metrics that emphasize overlap with the cardiac silhouette provide a more clinically meaningful assessment [11]. Prior reviews of medical image segmentation have cautioned against overreliance on pixel accuracy, highlighting its insensitivity to small but clinically significant differences in anatomical boundaries [12]. Overlap-based metrics such as Dice and IoU are therefore more appropriate for evaluating tasks like cardiac silhouette segmentation, where precise contouring is critical for downstream analysis.

Our findings are consistent with prior work showing that U-Net and its derivatives routinely achieve Dice scores above 0.90 in thoracic segmentation tasks [13], underscoring its adaptability across modalities. Li et al. (2022) found that U-net was able to segment lung imaging in chest X-rays (CXR) at high accuracy (Dice scores between 0.98-0.99 depending on the model used). But they found limitations in the processing of certain diseases that affect the boundary determinants by making the region more cloudy [13]. DeepLabV3, especially when paired with lightweight backbones such as MobileNetV2, has been reported to perform competitively in lung segmentation tasks [14], yet its performance in cardiac applications has been less extensively studied. In chest CT imaging, Murugappan et al. (2023) evaluated DeepLabV3+ using several feature extractors, including MobileNetV2, to segment both the lungs and COVID-19 lesions. They found that the model performed very well for whole-lung segmentation (IoU scores around 0.96-0.98), demonstrating that its ability to capture information at multiple image scales helps it identify large, well-defined structures [14]. By placing both architectures under identical conditions in the present study, we provide new evidence that U-Net retains an edge for cardiac silhouette segmentation.

Clinically, accurate segmentation of the cardiac contour has direct implications for calculating cardiothoracic ratios, detecting cardiac enlargement, and supporting longitudinal monitoring in both inpatient and outpatient settings. Cardiothoracic ratios are traditionally defined on chest radiographs as the ratio of the transverse heart dimension to the transverse chest dimension. These ratios have been found to be useful prognostic indicators, being positively associated with higher risk for cardiovascular complications and death. Accurate measurements are clinically important because threshold cutoffs guide provider decision-making; while >0.5 on PA CXR is traditionally considered cardiomegaly [15], CT data indicate that 0.56, 0.59, and 0.60 better predict differing degrees of systolic dysfunction, meaning that small boundary errors can alter interpretation [16]. Beyond cardiothoracic ratio estimation, automated segmentation of the cardiac contour could facilitate large-scale retrospective studies, support automated flagging of cardiomegaly in screening programs, and provide input features for multimodal AI models that integrate clinical, radiographic, and echocardiographic data. Thus, reliable silhouette segmentation represents a foundational step toward broader AI applications in cardiovascular imaging.

Future work should address three directions: (1) validating performance across external and multi-institutional datasets to assess generalizability; (2) extending segmentation granularity from the global cardiac silhouette to individual chambers and great vessels to increase clinical relevance; and (3) benchmarking against newer families of architectures, such as hybrid U-Nets and transformer-based models, which may capture both local boundaries and long-range dependencies. Such efforts will be necessary before these methods can be translated into routine use.

Several limitations should be considered when interpreting these findings. First, our study was conducted on a single public dataset of 1717 cardiac catheterization radiographs without patient or study identifiers, preventing us from ensuring that images from the same patient were exclusively assigned to a single data split, and external validation across multi-institutional cohorts is needed to assess generalizability to different imaging protocols, patient populations, and radiographic acquisition parameters. Second, we focused exclusively on binary segmentation of the global cardiac silhouette rather than more granular anatomical structures such as individual cardiac chambers or great vessels, which would provide greater clinical utility for specific diagnostic applications. Third, our comparison was limited to two architectures - U-Net and DeepLabV3 with MobileNetV2 - and did not include newer models such as transformer-based segmentation networks or hybrid architectures that combine convolutional and attention mechanisms. Fourth, we did not evaluate model performance across different cardiac pathologies or image quality degradations (e.g., portable radiographs, presence of implanted devices, or severe cardiac enlargement), which may affect segmentation accuracy in real-world clinical settings. Finally, while our evaluation metrics (Dice, IoU, and pixel accuracy) are standard in segmentation tasks, we did not assess clinical endpoints such as cardiothoracic ratio measurement error or agreement with expert manual annotations, which would be necessary to determine readiness for clinical deployment.

## Conclusions

This study compared the performance of U-Net and DeepLabV3 for cardiac silhouette segmentation on catheterization radiographs and demonstrated that U-Net achieved significantly higher Dice, IoU, and pixel accuracy scores, with large effect sizes confirming the robustness of this advantage. These results reinforce U-Net's role as a strong baseline architecture for medical image segmentation and suggest that its encoder-decoder design with skip connections remains particularly effective for tasks requiring precise boundary delineation. Although DeepLabV3 also produced clinically acceptable results, its relative underperformance underscores the importance of careful model selection for specific medical imaging applications.

Accurate segmentation of the cardiac silhouette carries direct clinical relevance for automated cardiothoracic ratio calculation, detection of cardiomegaly, and longitudinal monitoring, highlighting the potential of deep learning-based methods to enhance efficiency and reproducibility in future clinical integration. Future work should prioritize validation across diverse datasets and imaging environments, extend segmentation to individual cardiac chambers and vascular structures, and benchmark against newer architectures such as hybrid U-Nets and transformer-based models. Advancing along these directions will help move automated segmentation closer to becoming a reliable and scalable tool in routine cardiac imaging practice.

## Appendices

Link to external public cardiac dataset: https://www.kaggle.com/datasets/killa92/cardiac-semantic-segmentation-dataset
